# Informing Near-Airport Satellite NO_2_ Retrievals Using Pandora Sky-Scanning Observations

**DOI:** 10.1021/acsestair.4c00158

**Published:** 2024-11-13

**Authors:** Asher P. Mouat, Elena Spinei, Jennifer Kaiser

**Affiliations:** † School of Civil and Environmental Engineering, Georgia Institute of Technology, Atlanta, Georgia 30332, United States; ‡ NASA Goddard Space Flight Center, Greenbelt, Maryland 20771, United States; § School of Earth and Atmospheric Sciences, Georgia Institute of Technology, Atlanta, Georgia 30332, United States

**Keywords:** Aviation, airport, TROPOMI, NOx, vertical profile, MAX-DOAS, AMF

## Abstract

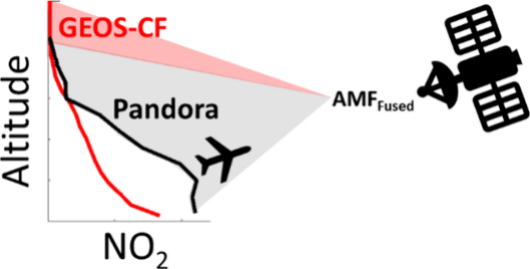

Airports are a large and growing source of NO_*x*_. Tracking airport-related emissions is especially difficult, as a portion of emissions are elevated above the surface. While satellite-based NO_2_ observations show hot-spots near airports, near-source retrievals often have large biases related to uncertainties in the NO_2_ vertical distribution and resultant air mass factors (AMF). Here we use observations from UV–vis spectrometers (Pandora 1S, SciGlob) deployed near the Atlanta Hartsfield-Jackson International airport from April 2020–May 2021 to assess the impact of aviation on NO_2_ vertical profiles. We show the first near-airport sky-scanning Pandora observations, which are used to distinguish the airport plume from the urban background. We find that increasing aviation leads to higher NO_2_ over the airport, and the enhancement is distributed across the mixed layer near-equally. We compare observed profiles with those modeled by the Goddard Earth Observing System composition forecast (GEOS-CF) system. We find that modeled profiles attribute a larger portion of the column closer to the surface and underestimate the NO_2_ mixing height. Observed profiles typically exhibited greater NO_2_ concentrations up to 2.5 km above ground level. Air mass factors (AMF) calculated using observations (AMF_Fused_) are similar over Hartsfield-Jackson to those calculated using GEOS-CF (AMF_GEOS-CF_). The unexpected similarity in alternative AMFs is attributed to the altitude-dependent sensitivity of AMF_Fused_ to changes in NO_2_ concentration. Using either AMF_Fused_ or AMF_GEOS-CF_ to evaluate TROPOMI NO_2_ against independent direct-sun observations produces consistent normalized mean differences of −22% and −29%, respectively. Overall, these results demonstrate the benefits of a combined ground and satellite-based approach for probing a complex distribution of NO_*x*_ emissions in an urban area.

## Abstract 2

Observational constraints on airport-related emissions are limited. We use 3D observations from the Pandora instrument to map the near-airport NO_2_ enhancement and discuss implications of discrepancies in measured and modeled NO_2_ vertical profiles for interpretation of satellite-based observations.

## 1 Introduction

Emissions of pollutants such as nitrogen oxides (NO_*x*_ = NO + NO_2_) and fine particulate matter from aircrafts, ground-support equipment, and airport-associated traffic negatively impact air quality and human health in near-airport communities.^1^ By 2045, international full-flight aircraft NO_*x*_ emissions are projected to increase between 140 and 340% relative to 2015 levels as demand for air travel rises.^2^ Near-airport observations are needed to evaluate emissions inventories and track the impact of aviation on local air quality. Several studies have demonstrated the use of new high-resolution satellite retrievals of NO_2_ in top-down estimates of emissions from urban areas and point sources.^3,4^ While these and similar studies outline promising methods for monitoring colocated emissions, interpreting near-airport retrievals is complicated by horizontal heterogeneity of NO_*x*_ concentrations and their potential emissions aloft. Validation of satellite products is an essential first step toward using satellite-based observations as constraints on at-airport emissions.

Pandora spectrometer systems (SciGlob) are particularly useful for satellite validation efforts given the similar measurement approach to satellite instruments, successful deployment during multiple field campaigns, and the growing international Pandonia Global Network (https://www.pandonia-global-network.org/, last access: 22 August 2024). For instance, total vertical column NO_2_ measurements from direct-sun (DS) measurements are of high quality and have been used in a number of satellite validation studies intensives.^5−10^ Subtraction of the stratospheric component determined from observations, models, or a fused model/observation component provide a DS tropospheric column that can be used for validation of satellite NO_2_ tropospheric columns,^9−11^ and more generally to examine the evolution of surface-to-column NO_2_ ratios throughout the day.^12^

Pandoras can also operate in sky-scanning mode to measure sky-scattered solar radiation in the desired azimuth viewing direction. Analysis of sky spectra using multiaxis differential optical absorption spectroscopy produces independent tropospheric vertical column densities (VCDs) and vertical profile estimation below 3–4 km (depending on the aerosol properties and conditions). Compared to DS observations, sky-scanning observations have higher sensitivity to lower altitudes and are therefore well-suited for examining near-surface emissions. The lower sensitivity to NO_2_ at high altitudes (>3 km) means that tropospheric VCDs from sky-scanning measurements will often underestimate the full tropospheric VCD. While the Pandora sky-scanning tropospheric column products are relatively new compared to the DS products, prior work has shown good agreement with other MAX-DOAS and aircraft observations^7,8,13^ and strong correlation with DS-derived tropospheric VCDs.^11^ Products from sky-scanning observations were evaluated during the CINDI-2 campaign and obtained the same features in the retrieved NO_2_ vertical distribution with a comparable root-mean-square deviation to other, established MAX-DOAS algorithms.^13,14^

Since August 2019, the TROPospheric Ozone Monitoring Instrument (TROPOMI) aboard the Sentinel-5 Precursor satellite has provided daily NO_2_ measurements with a flyover time of approximately 13:30 LT and a nadir spatial resolution of 3.5 × 5.5 km^2^. In polluted areas, the standard TROPOMI NO_2_ VCD product typically exhibits negative biases compared to ground- or aircraft-based observations.^6−9,15^ Discrepancies are often attributed to the use of horizontally coarse chemical transport models (CTMs) for generating a priori vertical profiles used in calculating air mass factors (AMFs). However, even fine-resolution models may not accurately capture the complex meteorological conditions, magnitude and distribution of emissions, and plume dynamics that influence near-source NO_2_ AMFs.^16−19^ Empirical NO_2_ vertical profiles can be used to identify systematic biases in the CTMs that are used to generate AMFs. Additionally, the use of empirical profiles in AMF calculations allows for evaluation of satellite products unaffected by CTM biases.

Launched in 2023, the TEMPO (Tropospheric Emissions: Monitoring of Pollution) instrument provides hourly daytime NO_2_ retrievals at a resolution of 2.1 × 4.4 km.^2,20^ A priori profiles from TEMPO retrievals are informed by the GEOS-CF (Global Earth Observing System Compositional Forecast) CTM, which operates at a horizontal resolution of 25 × 25 km.^2,21^ Vertical profiles from GEOS-CF and the related GEOS-Chem CTM have been shown to be particularly sensitive to modeled boundary layer heights and chemical mechanisms^16,22−24^

This study uses Pandora-1S and TROPOMI observations near the Atlanta Hartsfield-Jackson airport (ATL), the world’s busiest airport,^25^ to examine the impact of aviation on NO_2_ vertical profiles, and to demonstrate the benefits of using Pandora sky-scanning observations to calculate AMFs. Measurements were made from April 2020 to May 2021, which is a period that exhibits a large reduction in NO_*x*_ emissions as it contains part of the COVID-19 lockdown. Using TROPOMI data and a statistical plume-fitting approach, Fioletov et al.^3^ estimated a 55% decline in airport NO_2_ emissions during these periods (when passenger flights were down 75% and cargo down 25%) due to the pandemic. Additionally, they estimated emissions from ATL to be 5.1–6.4 kt yr^–1^ for 2018–2019, which is 40–73% higher than the 2017 EPA NEI estimate of 3.7 kt yr^–1^. Our observations provide a ground-based perspective and useful point of comparison for satellite-based assessments during a unique period.

In section 3.1, we use time series to demonstrate how changes in airport-activity and nearby on-road traffic impacted Pandora and TROPOMI VCDs. In section 3.2, we describe the Pandora DS and sky-scanning products and the impact of viewing geometry on agreement between measurements by comparing tropospheric VCDs derived from these data sets. In section 3.3, we compare Pandora sky-scanning and GEOS-CF vertical profiles and the impact of aviation on NO_2_ concentrations aloft. In section 3.4, we evaluate TROPOMI tropospheric NO_2_ VCDs with Pandora sky-scanning and DS retrievals, after recalculating AMFs using either GEOS-CF or empirically based profiles. We conclude with a discussion of the benefits and limitations of using TROPOMI observations for evaluation of CTM columns and emissions estimates for aviation and similarly complex urban sources.

## 2 Materials and Methods

### 2.1 Pandora Observations

The Pandora system is originally described in Herman et al.^26^ Briefly, the instrument optical sensor head collects photons in a narrow field-of-view (FOV = 1.5° at full width at half-maximum for sky-scanning and 2.6° for DS), which are then transmitted through a fiberoptics cable (single core, 400 μm in diameter) to a medium spectral resolution spectrometer (0.6 nm per full width at half-maximum). Solar spectra in the range of 280–525 nm can be measured at varying integration times (2.5 ms to 4 s) and averaged to improve the signal-to-noise ratio.^27^ The sensor head is mounted on a two-axis positioner which allows for observations in DS and sky-scanning geometries.

**Figure 1:**
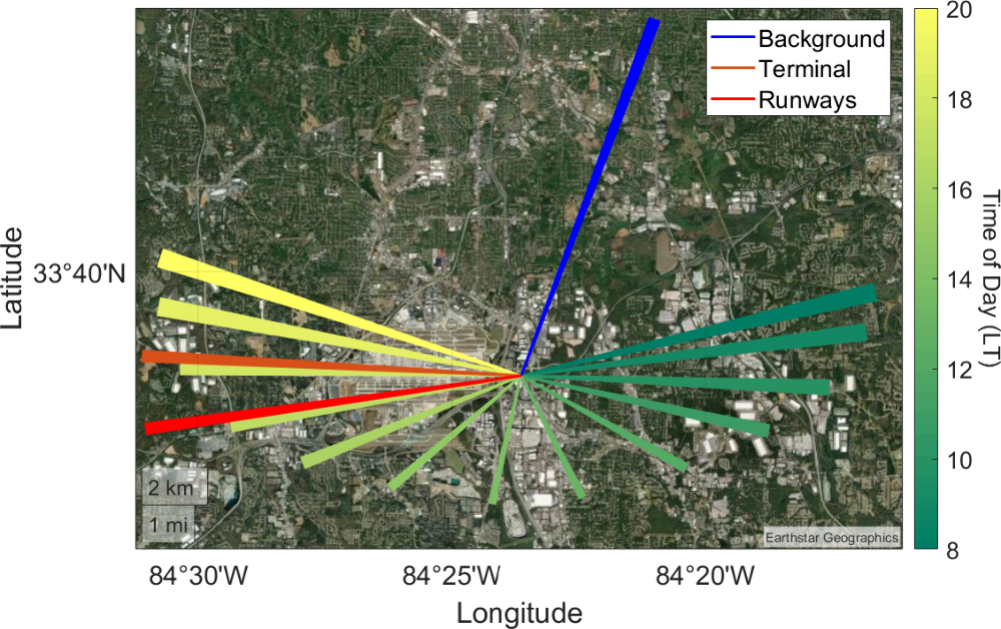
Pandora DS and sky-scanning scanning azimuth viewing directions. Line segments originate at the ground site and extend to the mean sky-scanning optical path length (11.4 km). DS lines of sight are colored by local hour of day and are capped to the same optical path length as sky-scanning measurements for purpose of visual clarity. FOVs for sky-scanning (1.5°) and DS (2.6°) are used to show a 2-D projection of the Pandora’s conical views. Base map from Esri World Imagery^28^ (reproduced with permission).

Two Pandora spectrometer instruments (Pandora 158 and 168, SciGlob, Ltd.) were deployed at the Georgia Environmental Protection Division Air Protection Branch offices, located 1.2 km east of the ATL runways (Figure 1), with an altitude above sea level of 291 m. Measurements began on 16 April 2020 and continued through 16 May 2021. Pandora 158 operated in DS mode while Pandora 168 alternated between DS and sky-scanning modes. Both instruments took measurements during daylight hours which were from 07:00–20:00 LT. Viewing azimuth angles (VAA) for sky-scanning observations targeted the urban background (VAA = 20.5°), the airport runways (VAA = 262°), and the terminal (VAA = 273°). There is an assumption that the air is heterogeneous between different azimuth directions within the remote sensing sampling volume (average photon path distance). Viewing zenith angles (VZA) of 2°, 50°, 60°, 65°, 70°, 75°, 80°, 81°, 82°, 83°, 84°, 85°, and 86° were used for all ATL-facing scans. For background scans, the lowest VZA was 85.5° and were otherwise the same.

The Pandora’s line of sight for each VAA is shown in Figure 1. Sky-scanning lines of sight are the horizontally projected optical path lengths calculated by BlickP, averaged over the full deployment period. They extend 11.4 km out from the instrument location. DS optical path lengths are colored by local time of day and are capped to the sky-scanning mean optical path length for visual clarity (DS path lengths are longer as their light source is more intense). DS VAAs start each day in the east, closer to the background direction, and end closer to the airport-facing direction. When VAAs of DS and sky-scanning measurements are most similar, the DS VZA is large (≥70°).

DS measurements were taken with integration times of 2.5 ms to 4 s and had a total measurement duration of 40 s. Sky-scanning measurements had an integration time of 200–300 ms (averaging over ≤20 s). This produces DS measurements from P168 averaged to a 1 min resolution and sky-scanning observations averaged over 7.5 min that occurred at each direction approximately every 30 min. The instruments produce 250 to 350 DS measurements per day, taking 20 to 30 observations each hour (the variability in number of hourly measurements results from the Pandora performing other routines throughout the day, e.g. sun-searching). Urban background absorption spectra were measured with an open filter wheel setting. Solar spectra were measured over ATL with a U340 optical filter (to reduce stray light in UV due to photons with wavelengths >385 nm) and without filters.

The standard Pandora sky-scanning viewing routine scans include measurements from the zenith to the lowest elevation angle, and back to the zenith at a single VAA, forming a “V-shape” in time. For runway and terminal VAAs, we scanned from zenith to the lowest level at one VAA, changed VAAs, and then scanned from the lowest angle to zenith. This allowed for greater measurement frequency. This viewing schedule is nonstandard and required postprocessing of the Pandora-generated L0 sky-scanning data for compatibility with the BlickP processing software. To create the requisite VZA symmetry, each vertical scan was duplicated to create the anticipated viewing pattern. While BlickP codes require observations at VZA > 87°, the tree line obscured the line of sight at angles >86°. We added artificial spectra at the expected VZAs of 88° and 89° but did not use them in the actual profile estimation. The lowest pointing angle (VZA = 86°) provided an average concentration within the lowest layer.

All spectra were processed using the BlickP software. Details of BlickP and r-codes can be found in the developer’s data products document.^29^ Total VCDs from DS measurements were obtained using BlickP v1.7.22 r-code “nvs3”, which uses the solar irradiance measured in the visible range of 400–440 nm. Retrievals of tropospheric and partial VCDs from sky-scanning measurements were obtained using BlickP v1.8.50 with r-code “nuh1”, (290–380 nm fitting window). Updates in Blick v1.8 are expected to have negligible impact on the total column product (which mainly result from the temperature treatment of molecular absorption cross section). Sky-scanning measurements are determined using the algorithm described in Frieß et al.^14^ (termed the “NASA real-time algorithm”). Briefly, the sky-scanning algorithm was developed by Elena Spinei Lind to provide real time estimation of the trace gas profile concentrations from spectroscopic radiance measurements while avoiding radiative transfer simulations (manuscript in preparation). It estimates trace gas profile shapes from absorption measurements of the trace gas and the oxygen collision complex (O_2_O_2_) normalized by the tropospheric column. The algorithm takes advantage of the fact that O_2_O_2_ absorption deviation from the Rayleigh case is due solely to aerosols and clouds. It then estimates the trace gas profile as the difference between the known O_2_O_2_ profile and the trace gas measurements. A detailed description of the algorithm can be found in Cede.^29^

We assign measurement uncertainties using the absolute uncertainties calculated by BlickP (“total” uncertainty for total VCDs, “independent” uncertainty for tropospheric VCDs). Detailed descriptions of the “total” uncertainty budget can be found in Spinei et al.^30^ and Cede.^29^ Uncertainties of the vertical profile concentrations are based on measurement error propagation through semiempirical equations, also found in Cede.^29^ Additional description of the sky-scanning algorithm, its uncertainty, and its QA process can be found in Rawat et al.^31^ (preprint).

For all Pandora data sets, we remove observations with solar zenith angles SZA > 75°, spectral fitting normalized rms weighted by uncertainty (wrms) > 0.0018 (approximately twice the median wrms in both geometries), and relative uncertainty >15%. We note that these criteria likely do not screen all cloud-affected measurements which generally exhibit greater uncertainty.^32^ We find that using highly restrictive data screening criteria (wrms <0.001, relative uncertainty ≤5%) does not affect normalized mean differences and improves correlation by ≤2% for TROPOMI tropospheric NO_2_ VCDs recalculated using alternative AMFs relative to our Pandora observations. Throughout this work, normalized mean difference (NMD) is used as a comparison metric. It is defined here as
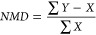
2where *Y* is any quantity relative to *X*, which is the point-of-comparison quantity. DS retrievals from the two Pandora instruments are well correlated (r = 0.98) and have a NMD of 2.4%. We therefore combine direct sun observations from both instruments into a single data set.

### 2.2 TROPOMI Retrievals

We use the offline level 2 v2.4 TROPOMI NO_2_ tropospheric and stratospheric products^33^ accessed at NASA’s Goddard Earth Sciences Data and Information Services Center (GES DISC, https://tropomi.gesdisc.eosdis.nasa.gov/). Data is filtered to exclude pixels with cloud fractions greater than 0.3, solar zenith angles greater than 60°, and quality flag (qa_value) less than 0.5. The VCD error provided in the product is taken as the measurement uncertainty.

This TROPOMI product uses TM5-MP modeled slant columns to separate its NO_2_ total slant columns into their stratospheric and tropospheric components. The TM5-MP model also provides the a priori vertical profiles used in the calculation of the TROPOMI tropospheric AMF (AMF_TM5-MP_). The procedure for updating AMFs based on alternative vertical profiles is outlined in Eskes et al.,^34^ which follows the approach of Palmer et al.,^35^ shown in eq 1.
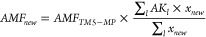
1where *AK*_*l*_ is the averaging kernel at level *l* (provided in the TROPOMI product), and *x*_*new*_ is the partial column in layer *l*. In this work, we use vertical profiles from GEOS-CF and a fused GEOS-CF/Pandora product to generate alternative AMFs for our TROPOMI evaluations. This procedure is described in the following section.

### 2.3 AMFs from GEOS-CF and Fused GEOS-CF/Pandora Profiles

GEOS-CF v1 model outputs^21^ are used to replace a priori vertical profiles in the TROPOMI product, to perform stratospheric separation for Pandora DS observations, and as a point of comparison with observed vertical profiles. The model is run on a cubed-sphere c360 horizontal resolution. The 72 hybrid-eta vertical grid results in a lower layer depth of approximately 130 m above ground level (agl) and a total of 14 model levels below 2 km agl. Anthropogenic NO_*x*_ emissions are from HTAP v2.2 which uses the 2008 U.S. EPA National Emissions Inventory,^36^ except for aviation-related emissions (ground support, landing and takeoff, and cruising) which come from the Aviation Emissions Inventory Code.^37,38^ Estimates of aircraft emissions are available for every calendar month in the year 2005. Emissions from both inventories are frozen to their respective years and are not adjusted for decreases in activity associated with the COVID-19 pandemic.

All TROPOMI retrievals shown in this work use AMFs generated from GEOS-CF vertical profiles (AMF_GEOS-CF_) unless otherwise noted. TROPOMI pixels are matched to the GEOS-CF hourly averaged 36-level replay output according to shortest distance between pixel and grid center points, and time bin containing the TROPOMI observation. Vertical remapping of GEOS-CF NO_2_ mixing ratios to TROPOMI scattering heights is performed assuming an exponential relationship between NO_2_ number density and pressure.

We find that at the location of the Pandora instruments, AMFs generated from GEOS-CF vertical profiles are well-correlated with the original AMFs (r = 0.70) but are lower with a NMD of −18.8%. Use of AMF_GEOS-CF_ increased TROPOMI NO_2_ VCDs at the location of the Pandoras by 25.4% compared to the original product. These results are anticipated given findings from prior studies that saw tropospheric VCD increases of 10–30% when using profiles from higher resolution CTMs (≥0.1° × 0.1°) to calculate alternative TROPOMI AMFs for retrievals measured over urban areas.^39,40^

**Figure 2:**
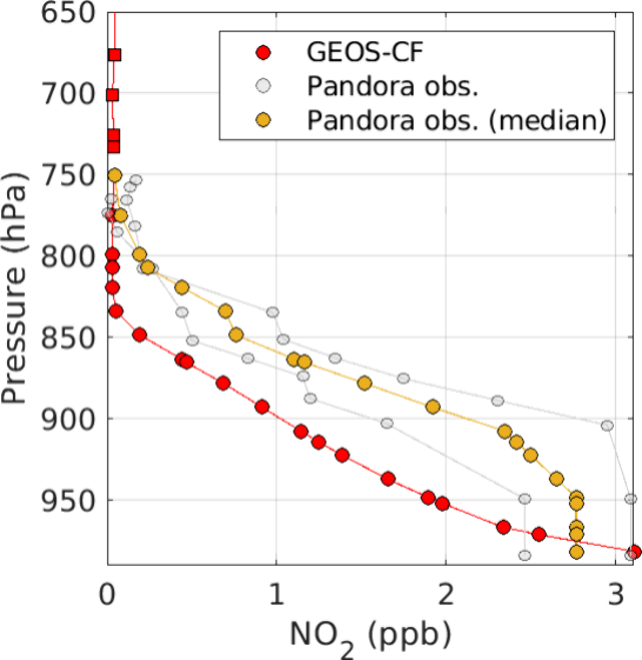
Example of vertical profiles measured over the ATL runways on 22 Mar 2021. Pandora observations have been interpolated to the GEOS-CF layer heights and are shown in gray. The resulting median profile used to partially replace the GEOS-CF profile is shown in gold. The replaced portion of the GEOS-CF profile is marked with circles and the segment that is unaltered is marked with squares.

We use Pandora sky-scanning vertical profiles to produce an observation/model fused AMF (AMF_Fused_). Figure 2 provides an example of this procedure using profiles from 22 Mar. 2021. For each TROPOMI overpass, we collect Pandora sky-scanning observations within 30 min of the TROPOMI overpass time which typically includes 2–3 vertical profiles per satellite observation. The concentration derived from the measurement taken closest to the horizon (VZA = 86°) is assumed to be constant down to the surface. Surface layer concentrations are expected to have high uncertainty. However, the corresponding TROPOMI averaging kernels (AKs) are smallest here and therefore have the least impact on AMF_Fused_ (discussed further in section 3.3). These profiles are then interpolated to the GEOS-CF NO_2_ layer heights assuming a linear relationship between mixing ratio and pressure (shown in gray). Following Dimitropoulou et al.,^15^ we calculate a daily median from these profiles (gold) to limit any instabilities in individual profile retrievals and replace the GEOS-CF NO_2_ vertical profile at the corresponding layer heights. The portion of the GEOS-CF profile to be replaced is shown as the red profile with circle markers. Additionally, Laughner et al.^18^ demonstrated the benefits of introducing daily variation into profiles used for calculating alternative AMFs. At altitudes where Pandora measurements are not available (>3 km agl), the original GEOS-CF profile is preserved (shown as the red profile with square markers). These new GEOS-CF/Pandora-fused shape profiles are used to generate AMF_Fused_ using eqn. 1.

### 2.4 Pandora DS Troposphere/Stratosphere Separation

We generate GEOS-CF/TROPOMI fused stratospheric VCDs from the hourly averaged replay product using an approach similar to that described in Adams et al.^12^ A polynomial equation is used to describe the hourly variation of stratospheric NO_2_ in the grid box containing the Pandora instrument over a 7-day moving average window. We apply the polynomial fits to the time point of Pandora DS observations to generate stratospheric VCDs on the same time basis as our observations. We then calculate the daily average ratio of TROPOMI stratospheric NO_2_ VCD observations to GEOS-CF modeled values. Gaps in the daily average ratio caused by days missing a TROPOMI-observed stratospheric VCD are linearly interpolated. The modeled stratospheric time series is multiplied by the daily average ratio to provide the GEOS-CF/TROPOMI fused stratospheric VCD. We find adjustments are typically minor (average TROPOMI/GEOS-CF stratospheric VCD ratio at the Pandora location = 0.97 ± 0.07). This model/observation fused stratospheric VCD is subtracted from the Pandora DS total NO_2_ VCD to produce a Pandora DS tropospheric VCD.

### 2.5 Pandora-TROPOMI Comparison Approach

Two Pandora data sets are either compared or combined with TROPOMI observations: the DS Pandora tropospheric VCDs and the Pandora sky-scanning tropospheric VCDs. For the DS comparison, all Pandora observations within ±10 min of the TROPOMI overpass time are averaged, with each Pandora observation weighted by the inverse of the reported total column measurement uncertainty. One data point is made for each TROPOMI observation that includes the Pandora location in its footprint.

For sky-scanning observations, spatial coincidence is determined using the method described in Dimitropoulou et al.^15^ For each VAA, we combine observations within 30 min of the TROPOMI overpass time. We draw a horizontal line segment in the direction of the VAA extending 5 km from the instrument location (Figure 1). This path length was chosen on the assumption that measurements made in UV are more representative of NO_2_ absorption within distances that are closer to and along the line of sight of the instrument. Sinreich et al.^41^ show that the probability of scattered photons reaching the instrument decreases exponentially with distance. Given the proximity of ATL to the instruments and that its area contains approximately 5 km of the Pandora line of sight for the runway and terminal directions, NO_2_ absorption beyond this distance is assumed to contribute negligibly. We produce a TROPOMI superobservation by combining the pixels through which the line segment traverses and weighting each TROPOMI pixel by the fraction of the line segment it contains. As in the DS comparisons, one sky-scanning data point is made for each corresponding TROPOMI superobservation.

### 2.6 Separation of High and Low Travel Periods

To examine the impact of aviation on our analysis, we identify two periods with similar meteorology, but varying levels of reported departures. Meteorological data are from an Automated Surface Observing System unit located at ATL (accessed at https://mesonet.agron.iastate.edu/ASOS/). The reported number of departures was manually recorded from www.flightradar24.com, accessed daily during the observation period. While the departure data does not encompass every flight, the relative change in observed departures is a relevant proxy for our analysis.

**Figure 3:**
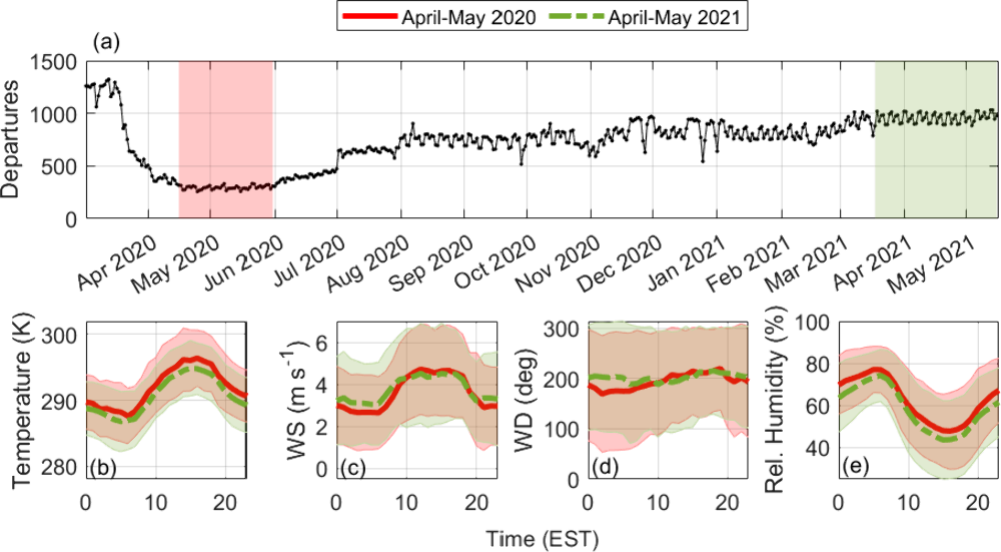
(a) Daily departures from ATL reported by Flightradar24.com. Shaded segments represent selected periods of high and low aviation-related activity with similar (b) temperature, (c) windspeeds, (d) wind direction, and (e) relative humidity. Shaded regions in (b)-(e) represent the standard deviation of the observations.

**Figure 4:**
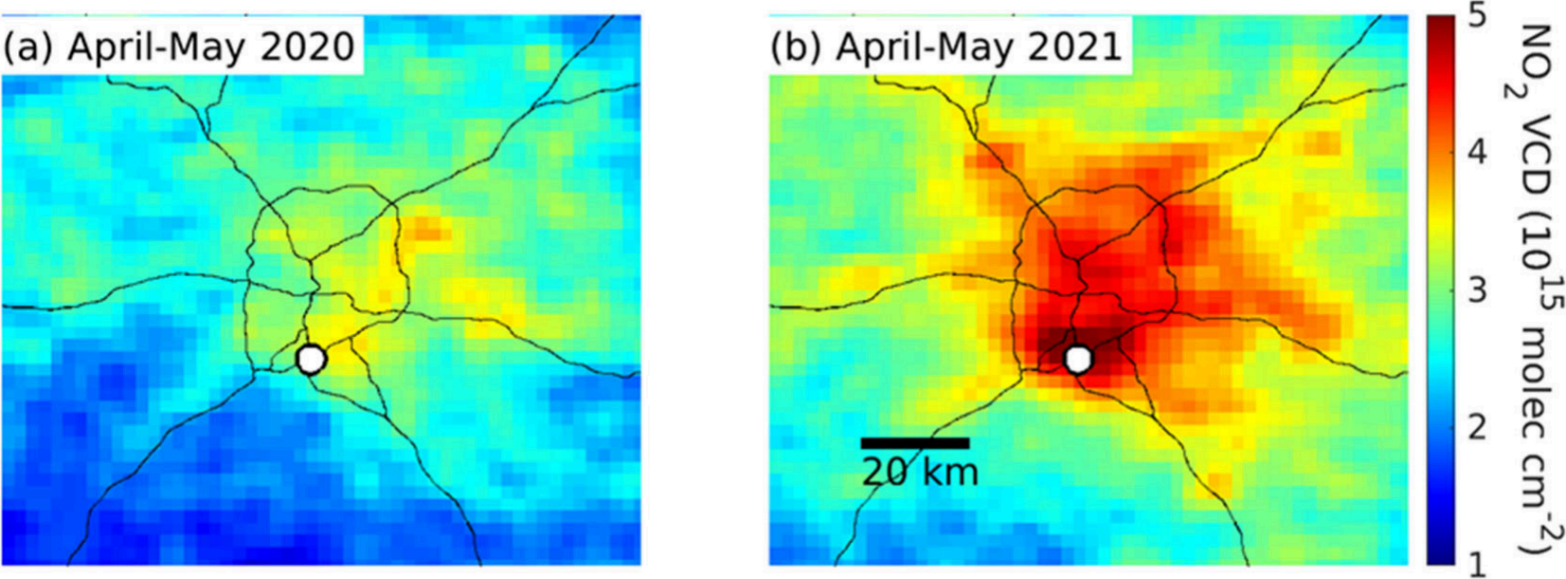
TROPOMI tropospheric NO_2_ VCDs for periods of (a) low and (b) high aircraft departures from ATL. VCDs have been oversampled to 0.02° using the method described in Sun et al.^42^ Lines denote major highways. The white circle is the measurement site, located immediately to the east of ATL.

Figure 3 shows the resurgence in aviation after spring 2020, and the time periods selected for comparison throughout our analysis. We select 16 April–31 May 2020 as our period of low aviation-related activity, with flightradar24 reporting an average of 295 departures per day. We identify 18 March–16 May 2021 as a meteorologically similar period with a much higher number of average daily departures (958). For simplicity, we refer to these periods as “April-May 2020” and “April-May 2021” in subsequent analysis. TROPOMI retrievals show a decrease in tropospheric NO_2_ VCDs throughout the entire Atlanta region during the April-May 2020 period (Figure 4). In the following sections, we discuss specifically the NO_2_ in the airport hot spot where the Pandora is located, and the impact of AMF calculations on this analysis.

## 3 Results and Discussion

### 3.1 NO_2_ VCD Variability

**Figure 5:**
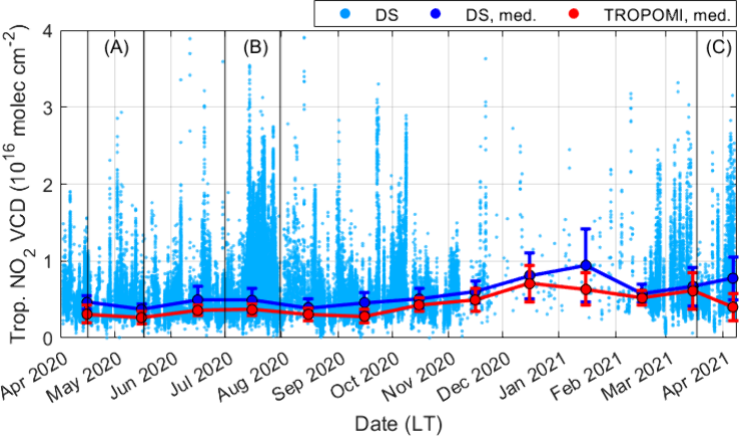
DS and TROPOMI tropospheric NO_2_ VCDs for the full deployment period. TROPOMI VCDs have been recalculated using AMF_GEOS-CF_. Monthly median VCDs are plotted for DS (blue) and TROPOMI (red) using data only from the TROPOMI overpass window (12–14 LT). Error bars are MADs. Three periods are delineated: (A) April-May 2020, (B) 1–31 July 2020, and (C) April-May 2021 and correspond to different levels of traffic and airport activity.

Time series for the full deployment period are plotted to demonstrate how changes in airport and traffic activity impacted DS and TROPOMI tropospheric VCDs (Figure 5). TROPOMI VCDs are recalculated using AMF_GEOS-CF_. Monthly midday median VCDs and their absolute deviations (MAD) are shown for DS and TROPOMI VCDs. DS medians and MADs are calculated using data measured from 12:00–14:00 LT. This period encompasses the range of TROPOMI overpass times that occurred throughout deployment (overpass time is not exact every day and TROPOMI may make more than one observation in a day that includes ATL). Three periods of interest are delineated: periods A and C are April-May 2020 and April-May 2021. Period B spans the month of July 2020, which exhibits an overnight increase of >154 daily departures on the first of the month.

Period A exhibits the lowest values and variability in VCDs with median DS and TROPOMI tropospheric VCDs of (4.2 ± 1.0) × 10^15^ molecules cm^–2^ and (2.7 ± 0.9) × 10^15^ molecules cm^–2^, respectively. During this period, urban on-road traffic counts were 50% lower than in 2019^43^ with a monthly average of 1.3 × 10^5^ counts (data from Georgia Department of Transportation, station 063–1192). Daily departures declined by 78% from the month prior (Figure 3). For period B, traffic volume plateaued (monthly average of 1.6 × 10^5^ counts, increase of 26% from period A), returning to business-as-usual levels. Daily departures increased by 117% relative to period A to a monthly average of 641 counts. Median and MADs for DS and TROPOMI VCDs increased to (4.9 ± 1.5) × 10^15^ molecules cm^–2^ and (3.7 ± 0.7) × 10^15^ molecules cm^–2^, respectively, which correspond to increases of 17% and 37%. We note that meteorological conditions have not been accounted for in these comparisons. Goldberg et al.^44^ determined that meteorology was favorable for low NO_2_ during spring 2020 across the U.S., so relative changes here may be exacerbated.

VCDs and variability are comparably greater for period C with median DS and TROPOMI tropospheric VCD values of (6.1 ± 1.9) × 10^15^ molecules cm^–2^ and (4.6 ± 1.7) × 10^15^ molecules cm^–2^, respectively (relative increases of 45% for DS and 71% for TROPOMI). The relative change in TROPOMI VCDs in this work is greater than what has been reported in prior studies, which determined decreases in tropospheric NO_2_ over ATL from 2019 to 2020 of 33–50% during spring.^3,43,44^ As these studies used VCDs with AMF_TM5-MP_, the higher relative change in VCDs in this work is attributed to use of AMF_GEOS-CF_.

Midday DS and TROPOMI monthly medians trend similarly throughout deployment. Both reach their minimums in May 2020 (DS: (3.7 ± 0.8) × 10^15^ molecules cm^–2^, TROPOPMI: (2.6 ± 0.8) × 10^15^ molecules cm^–2^) and maximums during December 2020 – January 2021 (DS: (9.4 ± 4.7) × 10^15^ molecules cm^–2^, TROPOPMI: (7.1 ± 2.4) × 10^15^ molecules cm^–2^). Daily departures continue to increase after winter, showing that the seasonal decrease in photoactive radiation enhanced NO_2_ lifetime. We note that DS observations are sparse throughout winter relative to the rest of the deployment period. Medians for December 2020 and January 2021 both include 30 measurements that temporally span each month. These medians trend similarly to the corresponding TROPOMI midday medians, lending confidence to their validity. Midday variability is also greatest during this period, though we note that DS MADs may be artificially inflated due to observational sparsity.

### 3.2 Impact of Pandora Viewing Geometry on Sky-Scanning Columns

**Figure 6:**
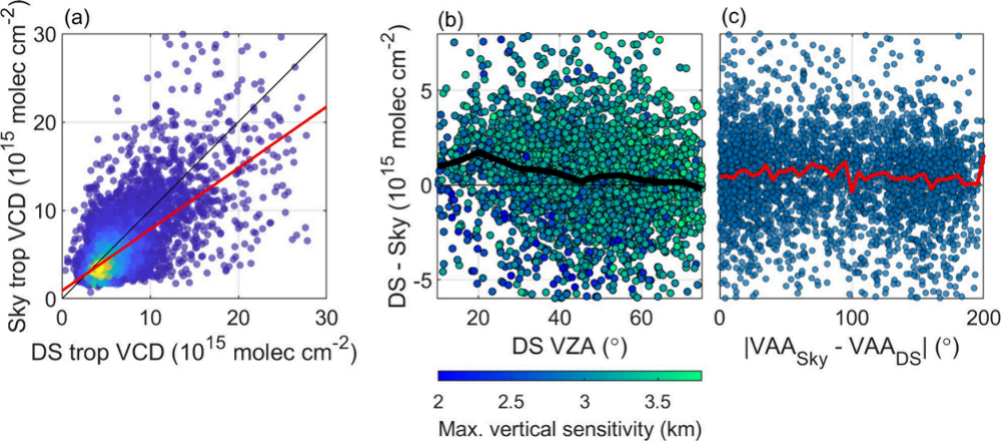
DS and tropospheric VCDs from the full deployment period. (a) compares sky-scanning and DS tropospheric VCDs using a York regression (red line) weighted by measurement uncertainty. (b) shows the difference between DS and sky-scanning tropospheric VCDs as a function of the DS VZA. The black line is the median VCD difference binned in 5° increments. (c) shows the difference in DS and sky-scanning VCDs (runway and terminal directions) as a function of the RAA. The red line is the median VCD difference binned in 5° increments. Data beyond the x and y range of the figure are not shown for clarity.

Pandora sky-scanning observations produce tropospheric columns that differ from DS observations for at least two reasons. The first is that DS and sky-scanning observations typically have different horizontal footprints (measurement direction and measurement surface projection, Figure 1). In Figure 6A, we compare DS and sky-scanning tropospheric VCDs under all viewing directions. The data sets correlate well with an r = 0.68. Sky-scanning observations are systematically lower than DS observations. A York regression,^45^ weighted using the uncertainties described in section 2.1 (DS tropospheric VCDs are assumed to have the same uncertainty as DS total VCDs), produces a slope of 0.69 ± 0.001 with an offset of (0.90 ± 0.01) × 10^16^ molecules cm^–2^. Sky-scanning tropospheric VCDs exhibit a NMD = −10.7% relative to the DS VCDs. These results are anticipated as sky-scanning measurements are more representative of the planetary boundary layer (PBL) and thus do not capture NO_2_ in the upper troposphere.^11,13^

Pinardi et al.^11^ performed a similar comparison for three different polluted cities, but calculated slopes greater than unity and intercepts that were negative. These discrepancies likely result from the different techniques used to apply a stratospheric correction to the DS data. Tropospheric VCDs reported in this work are less than or similar to those measured in the other cities of study (Xianghe, Beijing, and Thessaloniki), which suggests the PBL for the Atlanta area is less polluted. In more polluted regions, a greater amount of NO_2_ resides in the PBL, decreasing the contribution of upper tropospheric NO_2_ to the full tropospheric column. The subunity slope in this work could indicate that free tropospheric NO_2_ contributes notably to the full tropospheric column for the Atlanta area.

Differences between DS and sky-scanning observations are also found to reflect differing vertical sensitivities. Figure 6B shows the differences between DS and sky-scanning tropospheric VCDs as the DS VZA increases. These points are colored using the max vertical distance of the Pandora line of sight as a proxy for vertical sensitivity. The median difference at progressing DS VZAs shows a declining trend while the vertical sensitivity typically increases. As the DS VZA increases, DS vertical sensitivity toward tropospheric concentrations increases, and more closely approximates that of the sky-scanning viewing geometry.

Given our sky-scanning viewing geometry, ATL-facing measurements with small relative-to-sun azimuth angles (RAA = |VAA_Sky_ – VAA_DS_|) would be expected to agree better with DS columns as both VAAs and VZAs are similar (background RAAs are always large). Figure 6C shows the difference in DS and sky-scanning VCDs as a function of RAA. Median VCD differences are shown in red and do not exhibit a systematic relationship with RAA. This is attributed to two reasons: (1) Sky-scanning measurement integration times are 2 orders of magnitude longer than those of DS measurements. Light intensity increases as the instrument measures closer to the sun (RAA ≤ 30°) which can induce a “smearing effect”^46^ and bias the resulting VCDs. Measurements over ATL (which had westward-facing VAAs) could be subject to this in the afternoon. (2) even at small RAAs, high urban NO_2_ heterogeneity causes DS and sky-scanning geometries to not observe the same plume.

### 3.3 Impact of Aviation on NO_2_ Tropospheric VCDs and Vertical Profiles

**Figure 7:**
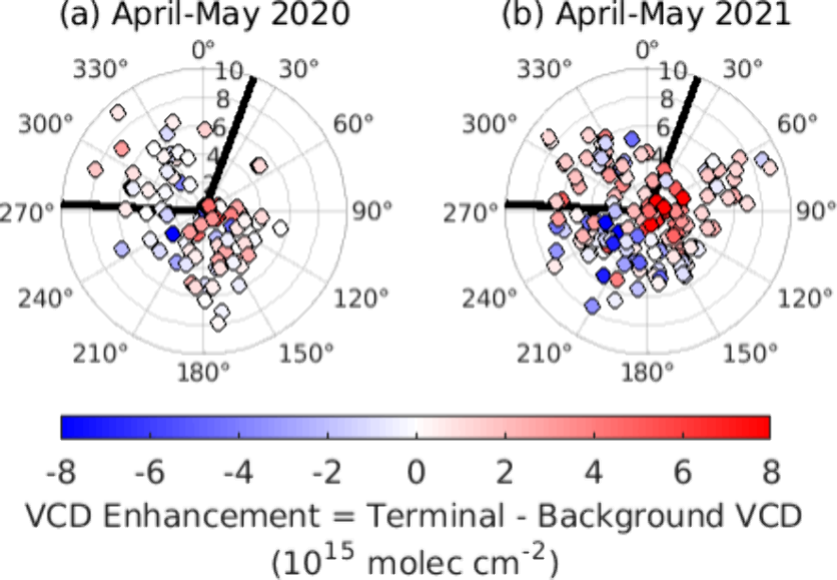
Midday (11–14 EST) enhancement of tropospheric VCD over the ATL terminal measured by Pandora sky-scanning observations as a function of windspeed and wind direction. Solid lines represent the VAA over the background (20.5°) and terminals (273°) under periods of (a) low and (b) high aviation.

The differences between NO_2_ tropospheric column densities measured over the airport reflect the prevailing wind patterns and the amount of airport activity (Figure 7). In both 2020 and 2021, enhancements over ATL are greatest when winds are westerly, and the background viewing direction is not influenced by emissions near ATL. Easterly and northeasterly winds transport near-airport emissions over the background viewing direction, leading to higher NO_2_ over the background than the airport. In April-May 2021, ATL enhancements are greater, with a mean increase from 1.88 to 2.86 × 10^15^ molecules cm^–2^ and an increase in daily median maximums from 2.49 to 4.42 × 10^15^ molecules cm^–2^. This is attributed to the increase in aviation relative to 2020. Background enhancements show a smaller mean enhancement increase of 1.49 to 1.68 × 10^15^ molecules cm^–2^ for the same periods.

**Figure 8:**
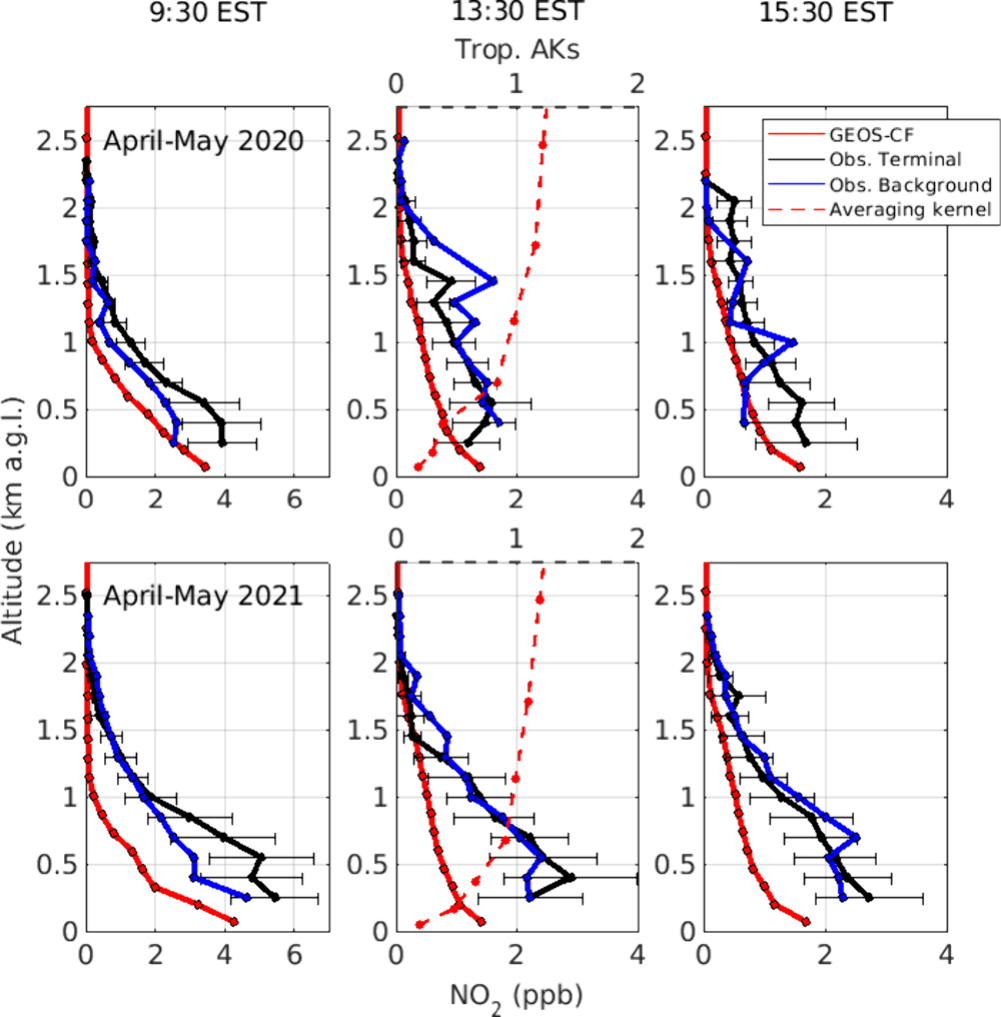
Median modeled and observed NO_2_ profiles for periods of low and high air traffic (top and bottom, respectively). Only observations with tropospheric VCD enhancements (= terminal-background) 3 times greater than the measurement uncertainty are used for constructing the median profile shapes. Error bars are the median absolute deviation at each altitude. The median averaging kernel profile is shown for the TROPOMI overpass time and corresponds to the dashed axis.

To examine the impact of aviation on NO_2_ vertical profiles, we isolate periods when Pandora sky-scanning observations are larger in the airport-viewing direction compared to the background, with the measured enhancement at least 3 times greater than measurement uncertainty. Figure 8 shows median observed vertical profiles (excluding the estimated surface layer) binned in 150 m altitude increments at different times of day for this subset of data. Points greater than 3 times the median absolute deviation of each bin are excluded. We show the background and terminal azimuth viewing angles only, as runway and terminal measurements produced similar profiles. The median absolute deviations for terminal and background-facing measurements are similar and are only plotted for the terminal profile.

In the morning (09:30 EST), airport vertical profiles consistently show elevated NO_2_ at altitudes ≤1 km agl compared to background. The difference between background and airport-facing vertical profiles is larger in 2021, when airport activity is higher. These enhancements are likely a result of the larger near-airport emissions in the second period and limited vertical mixing in the morning. By mid-day (13:30 EST or later), the observed profile shapes for the background and runway directions are more similar in both years. Vertical profiles over ATL continue to show slight enhancements at altitudes ≤0.5 km agl, but are reduced relative to 09:30 EST. The horizontal homogeneity in median mid-day profiles may reflect a higher relative importance of NO_2_ near the instrument and stronger atmospheric mixing. Absolute differences in overairport and background NO_2_ concentrations mid-day may be smaller than in the morning due to NO_2_ photolysis, and therefore more difficult to detect on top of any near-instrument NO_2_. These smaller absolute differences likely result from diurnal PBL growth and pollution mixing from extensive on-road and ATL sources which could result in a large, polluted air mass that potentially spans several km in radius around the measurement site.

Figure 8 also shows the median GEOS-CF vertical profiles for the grid cell containing the Pandora instruments. The profiles are consistent from year-to-year, as COVID-related impacts are not included in the model. As the boundary layer grows throughout the day, the NO_2_ mixing height increases, with sharp decreases in concentration around 1.25 km agl at 9:30 EST and near 2 km agl at 15:30 EST, and near-surface gradients becoming less pronounced. The observed shape profiles reflect an NO_2_ mixing height that is typically higher than modeled which is consistent with comparisons made between GEOS-Chem and either MAX-DOAS or aircraft vertical profiles from prior studies conducted in urban areas.^24,47^

### 3.4 TROPOMI NO2 Tropospheric VCDs: Impact of Modeled and Fused AMF

**Figure 9:**
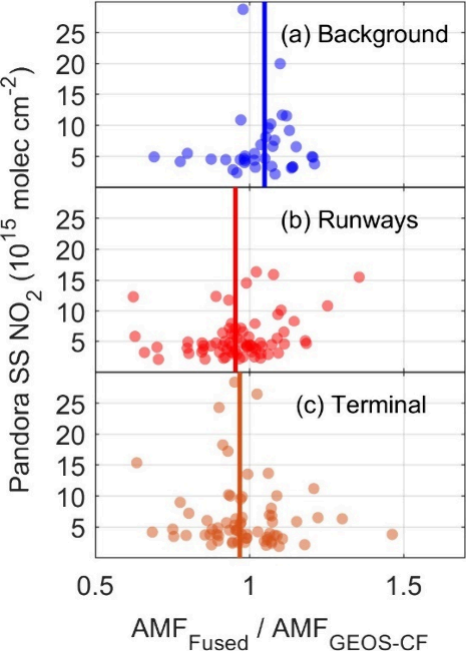
Ratio of AMFs calculated from fused and GEOS-CF vertical profiles for each Pandora viewing direction. Vertical lines indicate median values. For clarity, data points with ratios >2 are not shown (*n* = 0, 1, and 3 in panels (a), (b), and (c), respectively).

Figure 9 shows the ratio of AMF_Fused_ to AMF_GEOS-CF_ throughout the entire deployment period plotted against the corresponding sky-scanning VCD measurements. Median AMF_Fused_/AMF_GEOS-CF_ ratios are 1.05 (background), 0.95 (runways), and 0.97 (terminal). Sky-scanning tropospheric VCDs are typically greater than modeled and the fused profile has more NO_2_ nearer to the surface. Profile shape dictates the recalculated AMF values as they are normalized by the tropospheric VCD. Modeled profiles typically allocate approximately 87% (range of 80–95%) of the column within the first 0.75 km agl. By comparison, fused profiles in the runway and terminal directions allocate approximately 94% (range of 88–98%) within the same altitude, and would therefore be expected to produce smaller AMF than AMF_GEOS-CF_. However, while measuring over the North Sea, Riess et al.^48^ found a low sensitivity of recalculated AMFs to changes in near-surface concentrations, where a 50% increase in surface concentration resulted in only a 10% change in the AMF.

We perform similar sensitivity tests as in Riess et al.^48^ AMF_Fused_ were recalculated two ways: by doubling either 1) the surface concentration of the profile or 2) the concentration of the layer closest to 1 km agl. Doubling the surface concentration produced a linear regression between the recalculated and original AMF_Fused_ with a slope of 0.97. The same comparison after doubling the concentration at 1 km agl led to a slope of 1.04. These results indicate that increasing the surface layer concentration is offset by similar increases at 1 km agl. Median Pandora vertical profiles frequently have greater concentrations than the corresponding GEOS-CF estimates at altitudes up to 2.5 km agl (for instance, when measuring an aloft plume). Since AMF sensitivity to NO_2_ concentration increases with altitude, the fused profiles result in only a slight decrease in nonbackground AMFs relative to AMF_GEOS-CF_.

The variability in the ratio is largest over the runway, with standard deviation (σ) of 0.14. Standard deviations in the terminal and background direction are 0.12 and 0.11, respectively. Deviation from the median AMF ratio at large tropospheric NO_2_ VCDs may be indicative of plumes, with greater ratios indicating the plume is elevated above the surface. Additionally, aircraft departures are based on prevailing wind speed and direction. Behere et al.^49^ determined take-offs from ATL were primarily west-bound along the two inner landing strips to the north and south of the terminal. The runway-facing line of sight intersects with the southern landing strip and is anticipated to exhibit the most variability in NO_2_ VCDs. Deviations at lower tropospheric NO_2_ VCDs often reflect a lack of NO_2_ observed at the surface rather than a plume aloft and results in lower-than-median AMFs.

**Figure 10:**
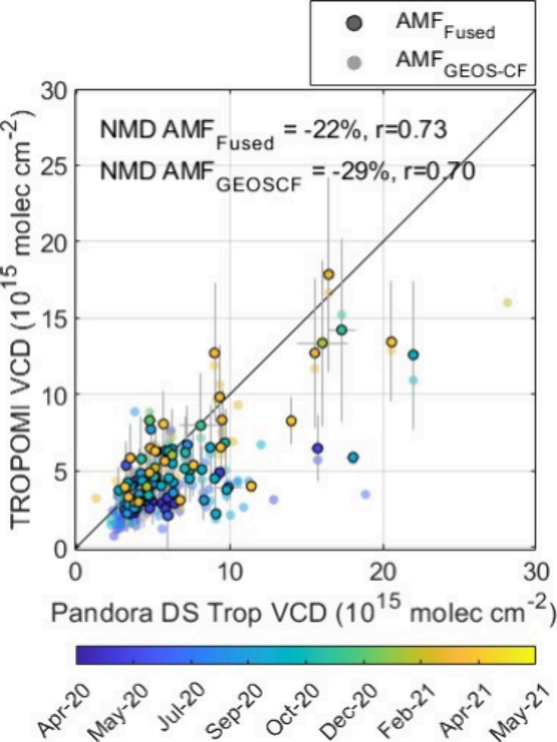
TROPOMI and Pandora DS NO_2_ tropospheric VCDs. TROPOMI products are updated using either AMF_GEOS-CF_ or AMF_Fused_. Error bars represent measurement uncertainty.

Using runway and terminal-facing AMF_Fused_ rather than AMF_GEOS-CF_ generally produces slightly greater TROPOMI tropospheric VCDs. Pandora DS observations provide an independent, high-precision tropospheric NO_2_ VCD data set for comparison. Results are shown in Figure 10. TROPOMI VCDs are superobservations in the runway direction, which is most similar to the Pandora DS footprint at the time of TROPOMI overpass (Figure 1). Correlations and NMDs are calculated as TROPOMI VCDs relative to Pandora VCDs. TROPOMI and Pandora DS tropospheric VCDs are well correlated with r = 0.70 for VCDs calculated using AMF_GEOS-CF_ and r = 0.73 for VCDs calculated using AMF_Fused_ over the entire deployment period. VCDs using AMF_GEOS-CF_ were determined to have a NMD = −29%, while VCDs using AMF_Fused_ produced a NMD = −22%. These biases are lower than Phaka et al.,^47^ who compared similarly recalculated TROPOMI VCDs (using daily median MAX-DOAS vertical profiles in an urban area) to MAX-DOAS tropospheric VCDs. They reported biases of −2.3% when comparing daily measurements and −11.5% for monthly averaged values. Correlations for monthly averaged data (r = 0.68) were comparable to correlations determined in this work. NO_2_ vertical profiles reported in their study indicate that our surface concentration assumption underestimates the actual concentration. Our results indicate that combining GEOS-CF or fused profiles with TROPOMI retrievals improves accuracy but still produces underestimated columns when monitoring airport NO_2_.

**Figure 11:**
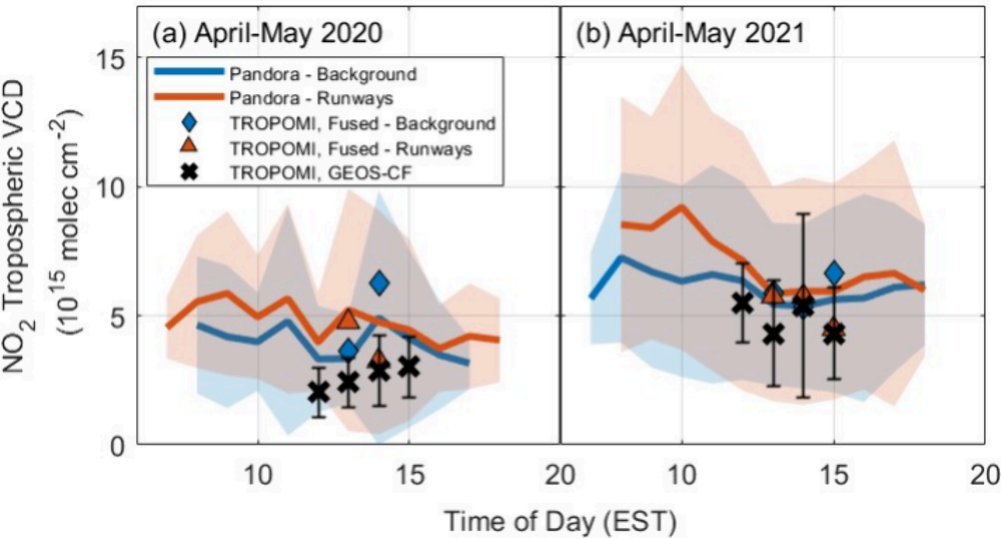
Hourly averaged Pandora sky-scanning VCDs overlooking the background or ATL runways during periods of (a) low and (b) high aviation. Shaded regions and error bars are the standard deviation of hourly averaged data. TROPOMI VCDs are calculated using AMF_GEOS-CF_ and AMF_Fused_. TROPOMI VCDs using AMF_Fused_ superobservations along the line of sight.

Looking forward to TEMPO, we examine diurnal variation in tropospheric NO_2_ VCDs over the runway and the airport and compare to the variation seen by TROPOMI, while noting that number of observations in the late afternoon is small. Observations for periods of high and low airport activity are shown in Figure 11. As also suggested by the vertical profiles in Figure 8, midafternoon Pandora sky-scanning tropospheric VCDs show little difference between viewing directions in both 2020 and 2021. In 2020, observations remain flat throughout the day, whereas 2021 shows overairport enhancements in the morning. From Figure 5, DS observations reveal that these morning enhancements begin in July 2020 when vehicle traffic stabilized and daily departures see a notable increase and continue throughout deployment. This suggests that satellite-based observations in the early morning will more clearly reflect the impact of aviation on near-airport emissions than what is possible with TROPOMI. These early morning profiles, however, may be more sensitive to errors in modeled mixing heights. We find that TROPOMI VCDs calculated using AMF_GEOS-CF_ are within the range of Pandora observations and show similarly small hourly variation in midafternoon. TROPOMI VCDs calculated using AMF_Fused_ show larger variation, which is a reflection both of greater observed NO_2_ variability and a more limited number of Pandora sky-scanning observations in this data set.

### 3.5 Implications

We report the first Pandora sky-scanning vertical profiles measured in a complex, heterogeneous environment. We find that using fused vertical profiles to inform TROPOMI AMFs results in a product that exhibits less bias than using only modeled profiles relative to independent Pandora DS tropospheric NO_2_ observations. However, both products still exhibit low biases indicating that alternative AMFs calculated in this work are too large. This is attributed to the resolution of GEOS-CF, underprediction in current day aviation-related NO_2_ emissions, Pandora measurements not directly measuring near-surface, and the assumption for our “observed” surface concentration being too low. The improvement in bias using fused profiles nonetheless gives confidence in the ability to use NO_2_ vertical profile estimates and observations from the rapidly expanding Pandonia global network not just for satellite evaluation, but for development of improved products. We note that results here using our unique viewing configuration and location may differ from studies in other environments and with the standard Pandonia viewing schedules. There is still a need for independent evaluation of Pandora sky-scanning observations using in situ observations.

We find that difference between observed and modeled vertical profiles, and between vertical profiles in different viewing directions, is largest in morning hours. This underscores the opportunity of observations from geostationary instrumentation like TEMPO to diagnose emissions at times when “hot-spots” appear brightest. Our results suggest that a correct representation of vertical mixing heights is especially important when using models to calculate a priori vertical profiles. In contrast, variability in observations suggest that even very different emissions scenarios such as those driven by the COVID pandemic have little impact on AMFs calculated from observations. This gives confidence in top-down emissions derivation methods that assume a constant emissions/AMF relationship. However, Jin et al.^50^ found that using GEOS-CF NO_2_ vertical profiles for calculating alternative TROPOMI AMFs led to enhancements in satellite-derived NO_2_ emission factors. Coupled with the agreement between the modeled and fused versions of TROPOMI VCDs and observations in this work, this indicates that prior emissions estimates like that of Fioletov et al.,^3^ who used the standard TROPOMI NO_2_ VCD product, may underpredict absolute emissions in urban areas or around point sources.

Further quantification of free tropospheric NO_2_ will be required. Dang et al.^16^ predicted a 14% increase in AMFs over the next decade, owed to increasing free tropospheric NO_2_ resulting from aircraft emissions. However, results from recent works show the difficulty in accurately modeling free tropospheric NO_2_.^16,23^ Pandora spectrometers have been used previously for measuring upper tropospheric NO_2_^51^ and could be beneficial to future modeling efforts. We note that the GEOS-CF profiles used in this work may underestimate free tropospheric NO_2_ which is impacted both by the choice of chemical mechanism^16,24^ and method of accounting for aviation emissions (for instance, use of the Aviation Emissions Inventory Code which is not scaled to current day emissions) As Pandora vertical profiles are representative of the boundary layer, TROPOMI tropospheric VCDs in this work are anticipated to underestimate NO_2_ in the upper layers regardless of AMF choice.

A prior work by Lawal et al.^52^ focused on NO_2_ emissions at ATL using a high resolution (4 × 4 km^2^) CTM coupled with a landing and takeoff emissions inventory to develop new AMFs. Updated TROPOMI VCDs saw a 60% increase over ATL compared to the standard VCD product, which is greater than the corresponding increases seen in this work when using AMF_GEOS-CF_ (25.4%) or AMF_Fused_ (17%). They noted improvement in bias between satellite and modeled VCDs, which was attributed to less steep, modeled NO_2_ vertical profiles near the airport due to the dilution of emissions. GEOS-CF vertical profiles in this work exhibit similar behavior, having a much steeper vertical gradient than observed, leading to underestimations of near-airport NO_2_ VCDs.

Even after correcting for the vertical profiles of NO_2_ observed over the airport, TROPOMI retrievals suggest that ATL is a dominant source of NO_*x*_ emissions in the Atlanta region. As is true for several airports, there are no regulatory monitors near ATL. Aviation-related emissions have a disproportionate impact on historically disadvantaged communities. Our results suggest that satellite observations coupled with higher resolution models provide a reliable starting point for diagnosing longer-term trends in NO_2_ and the underlying emissions from the growing airport source sector.
